# Prospective study of the application of a 3D exoscope system (VITOM 3D) in ear surgery compared to conventional surgical microscopes: part I - analysis of objective parameters

**DOI:** 10.1007/s00405-024-09096-9

**Published:** 2024-11-22

**Authors:** Hai Yen Tao, Joseph Morgenstern, Christoph Müller, Nikoloz Lasurashvili, Thomas Zahnert, Thomas Beleites, Marcus Neudert

**Affiliations:** Department of Otorhinolaryngology, Head and Neck Surgery, Ear Research Center Dresden, Technische Universität Dresden, Faculty of Medicine and University Hospital Carl Gustav Carus, Fetscherstraße 74, 01307 Dresden, Germany

**Keywords:** Middle ear surgery, Ear surgery, Exoscope, Vitom, VITOM, Cochlear implant, Cholesteatoma, Cholesteatoma surgery, Chronic oititis media, Tympanoplasty, Outcome, Outcome parameter, Objective parameter, Measurements, Intraoperative evaluation, Surgical assist

## Abstract

**Background:**

This prospective study compared the application of a 3D exoscope (VITOM 3D) with surgical microscopes (SM) in ear surgery.

**Methods:**

62 patients were included (exoscope group (E+) *n* = 31; SM group (E-) *n* = 31). Procedures included cochlea implantation (nE + = 10, nE- = 10), reconstructive middle ear surgery due to chronic otitis media with (COMwC; nE + = 11, nE- = 11) and without cholesteatoma (COMsC; nE + = 10, nE- = 10). Objective (e.g. set-up time and wrap-up time, corrected cut-suture-time, pure operating time, adjustment time, time per adjustment procedure, learning curves) and subjective perioperative parameters were evaluated. This paper only addressed the analysis of the objective parameters.

**Results:**

The pooled data showed that the mean time delay in the E + group was significantly higher compared to the E- group with regard to set-up time and wrap-up time, corrected cut-suture time, adjustment time and time per adjustment procedure. Time delay tended to be higher for pure operating time. In all subgroups of the E + group, the objective time parameters also showed at least a tendency to be prolonged on average compared to the corresponding subgroups in the E- group. The learning curve analysis showed that the E + group (corrected cut suture time for CI surgery, mean time per adjustment procedure across all subgroups) approached the times of the E- group during the course of the study.

**Conclusions:**

Based on the pooled data from the study arms, the exoscope tends to be inferior to the microscope for the objective time parameters evaluated when used in ear surgery. However, due to the small group sizes, no solid conclusions could be drawn regarding the individual surgical procedures. In addition, further studies with a longer observation period are needed to minimize the influence of the learning curve on the results.

**Supplementary Information:**

The online version contains supplementary material available at 10.1007/s00405-024-09096-9.

## Introduction

The surgical microscope (SM) has been the gold standard in ear surgery since its introduction by Wullstein in 1953 [1, 2]. Since the 1990s, exoscopes have been introduced as new intraoperative visualization systems. An exoscope consists of a camera positioned above the surgical field, for example by means of a manually moveable (passive holder) or telemanipulated (active holder) articulated arm [3]. The 2D or 3D image is transmitted to a monitor placed directly across the surgeon [4]. A main difference between the SM and the exoscope is the heads-up approach. The surgeon and operating room (OR) staff have an eye-level view which may help posture and reduce pain [5] and therefore improve performance of the surgery.

In the literature, the application of exoscopes in ear surgery has already been evaluated in skull base surgery and resection of vestibular schwannoma [6–8], transmastoidal cholesteatoma surgery [8–11], tympanoplasty [10–12], cochlear implantation [12, 13] and stapes surgery [10, 14]. Advantages of the exoscope were seen in the better ergonomics [7, 8, 12], ease of positioning [8] and educational aspects due to the shared use of the monitor [7, 8, 10–12]. Main points of criticism were the reduced image quality due to poor illumination [8] causing difficulties in differentiation between various tissues [7, 10, 12], as well as the decreased depth perception [6, 7]. The following exoscopes were used in the above mentioned studies: the VITOM 3D (Karl Storz GmbH, Tuttlingen, Germany) [7–14], the ORBEYE (Olympus Corporation, Tokio, Japan) [8] and the BrightMatter Servo respectively Modus V (Synaptive Medical, Toronto, Canada) [6].

However, the predominant study quality is not satisfactory. Many studies attempt to evaluate the advantages and disadvantages of using exoscopes versus microscopes, but often lack balance and randomization in the study arms, compare different interventions, and incompletely record objective and subjective parameters. The aim was therefore to address these shortcomings and to evaluate the advantages and limitations of the application of an exoscope (VITOM 3D, Co. Karl Storz GmbH, Germany) in comparison to surgical microscopes by means of defined objective and subjective parameters for three procedures in ear surgery with different degrees of standardization in a structured multi-arm randomized prospective study. The results of the objective parameters are presented in this paper, the results of the subjective parameters will be part of a future paper.

## Materials and methods

### Ethics approval statement

The study followed the tenets of the Declaration of Helsinki and was approved by the Institutional Review Board (IRB00001473) at the Technische Universität Dresden (EK 393102018).

### Study design

A prospective randomized study was conducted. The study consisted of two arms for the visualization systems: exoscope study arm (E+) and SM study arm (E-), respectively. Additionally, each arm consisted of three subgroups representing surgeries with different degrees of standardization: stapes surgery, cochlea implant (CI) surgery, and reconstructive middle ear surgery for chronic otitis media with cholesteatoma (COMwC). Due to a change during the study, stapes surgeries were excluded and replaced by reconstructive middle ear surgery for chronic otitis media without cholesteatoma (COMsC). All procedures were performed in a tertiary referral hospital between October 2019 and September 2020 and under general anesthesia.

The sample size of this study is based on the assumption of an actual difference in cut-suture time between the two methods of 10.0%. Assuming a clinically acceptable increase in cut-suture time of 20.0%, a Type-I error rate of α = 0.025, and a power of β = 0.9, the sample size is calculated as *n* = 27 per study arm, totaling *n* = 54, according to Julious [15], to adequately test the research hypotheses in the context of a non-inferiority study. Therefore, 62 patients (28 females and 34 males) between 18 and 84 years (mean age 53.4 ± 18.7 years SD (standard deviation)) with an indication for the above-mentioned procedures were enrolled after providing a declaration of consent. Individuals under the age of 18 were excluded from the study. After the first 8 procedures had been assigned by means of simple randomization to one of the two study arms and had been accomplished, a technical defect occurred on the exoscope. Therefore, it was required to switch to an en-bloc randomization and to assign all procedures to the E- study arm. After the targeted number of cases was reached in this arm and the technical defect of the exoscope was fixed, all remaining 27 procedures of the E + study arm were performed.

Finally, each of both study arms included 31 surgical procedures: 10 times CI surgery, 11 times reconstructive middle ear surgery due to COMwC and 10 times reconstructive middle ear surgery due to COMsC. Please refer to Annex 1 in the appendix regarding the demographic details of the patients.

The procedures were conducted by seven surgeons with two senior physicians (surgeon 301 and 302) performing more than 75% of all cases (48 out of 62; 77.4%).

### Operating room setup

Surgery in the E- study arm was conducted by means of a Pentero 800 (*n* = 18), an OPMI Vario 700 (*n* = 10) and a Vario S88 (*n* = 3) (all ZEISS, Oberkochen, Germany). Surgery in the E + study arm (exoscope) was performed by means of a VITOM 3D (Karl Storz GmbH, Tuttlingen, Germany).

**Fig. 1:**
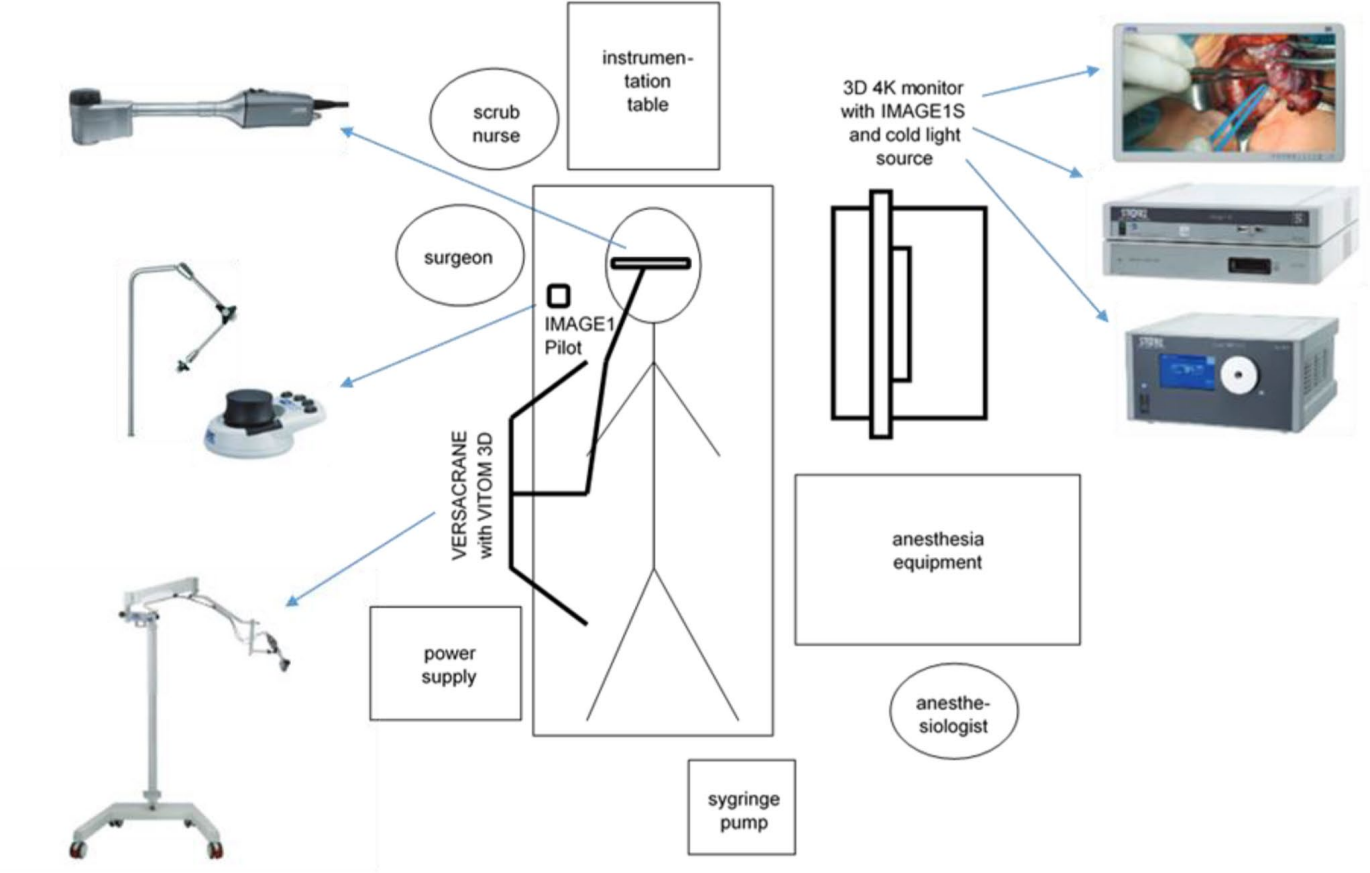
Operating room set up when operating the right ear with the VITOM stratified in their individual components; images of VITOM 3D, IMAGE1 PILOT, Versacrane, 3D Monitor, IMAGE1 S and cold light source provided by KARL STORZ SE & Co. KG

The surgical set-up is shown in Fig. 1 [16] and varied depending on whether the surgery was performed on a left or a right ear.

### Outcome parameters

**Fig. 2:**
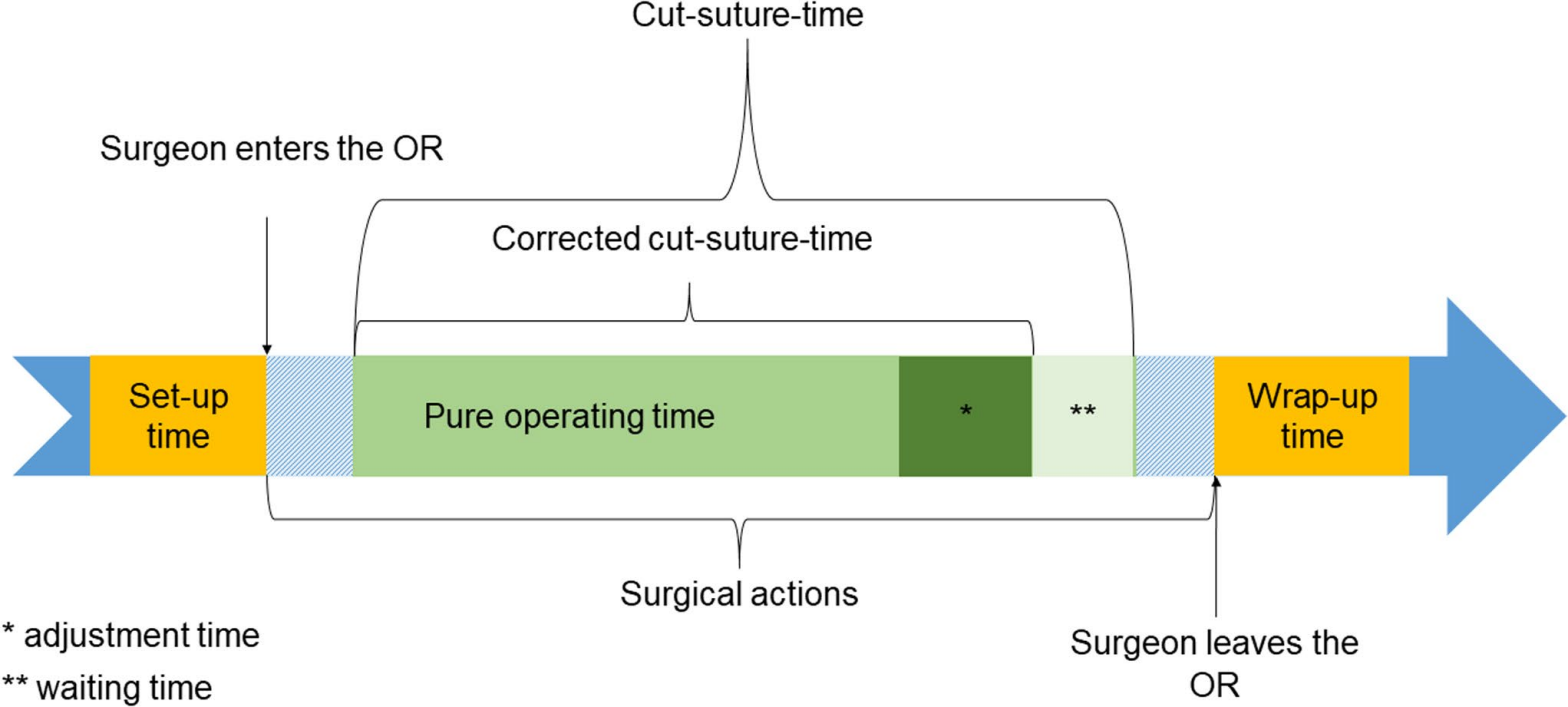
Temporal sequence of intraoperative and perioperative times. The times within the cut-suture time have been shown one after the other for simplicity, although they are mixed in the temporal sequence

Perioperatively, the times for set-up and dismantling were measured. Intraoperatively, each single step of the procedure was tracked using a customized stopwatch programmed in Microsoft Excel (Microsoft Inc., Redmont, USA) for determination of cut-suture-time (CST), time per and number of adjustment procedures of the visualization system, and disturbance or waiting times. From these data, the **corrected CST (cCST)**, the **pure operating time (POT)**, the **adjustment time per surgery**, the **mean time per adjustment procedure** and the **mean proportion of the adjustment time to cCST** were calculated. The measured and calculated times are visualized in Fig. 2.

In addition to these objective outcome parameters, subjective parameters were also assessed. This includes questions about the optical display quality for specific anatomical landmarks relevant to various surgical procedures. However, these will be discussed in the second part of this publication series.

### Learning curves

**Fig. 3:**
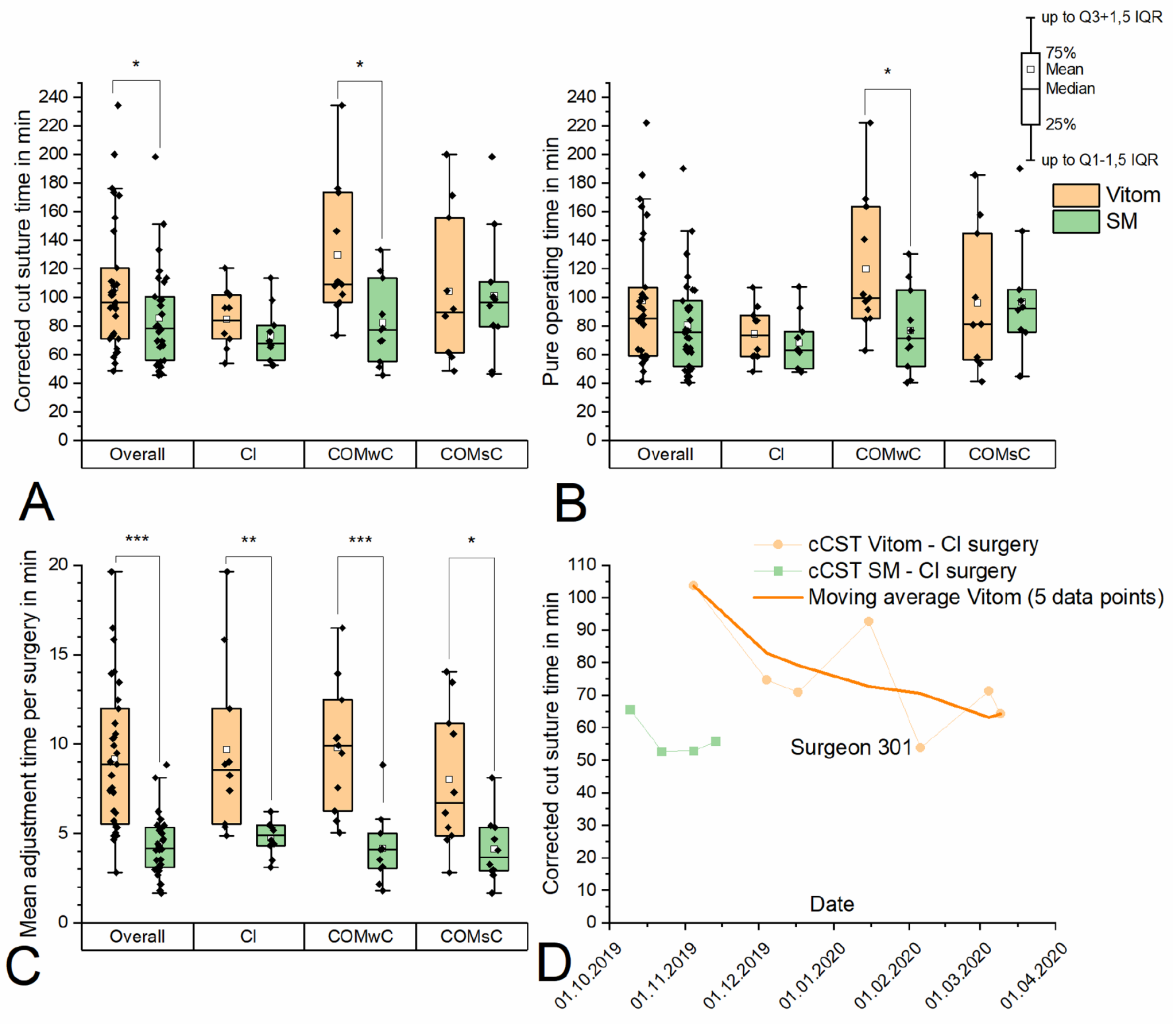
**A** Corrected cut suture time per procedure: significant prolongation with the VITOM in the pooled data set (‘overall’) and the subgroups COMwC and COMsC. **B** Pure operating time per procedure: significant prolongation with the VITOM in the pooled data set and the subgroups COMwC and COMsC. **C** Mean adjustment time per surgery: significant prolongation with the VITOM in all groups including the subgroups. **D** Learning curve of surgeon 301 for CI surgery with the VITOM based on the parameter ‘cCST’ compared to the use of the SM. The moving average indicates a learning progress with the VITOM with decreasing cCST over the course of the study

**Fig. 4:**
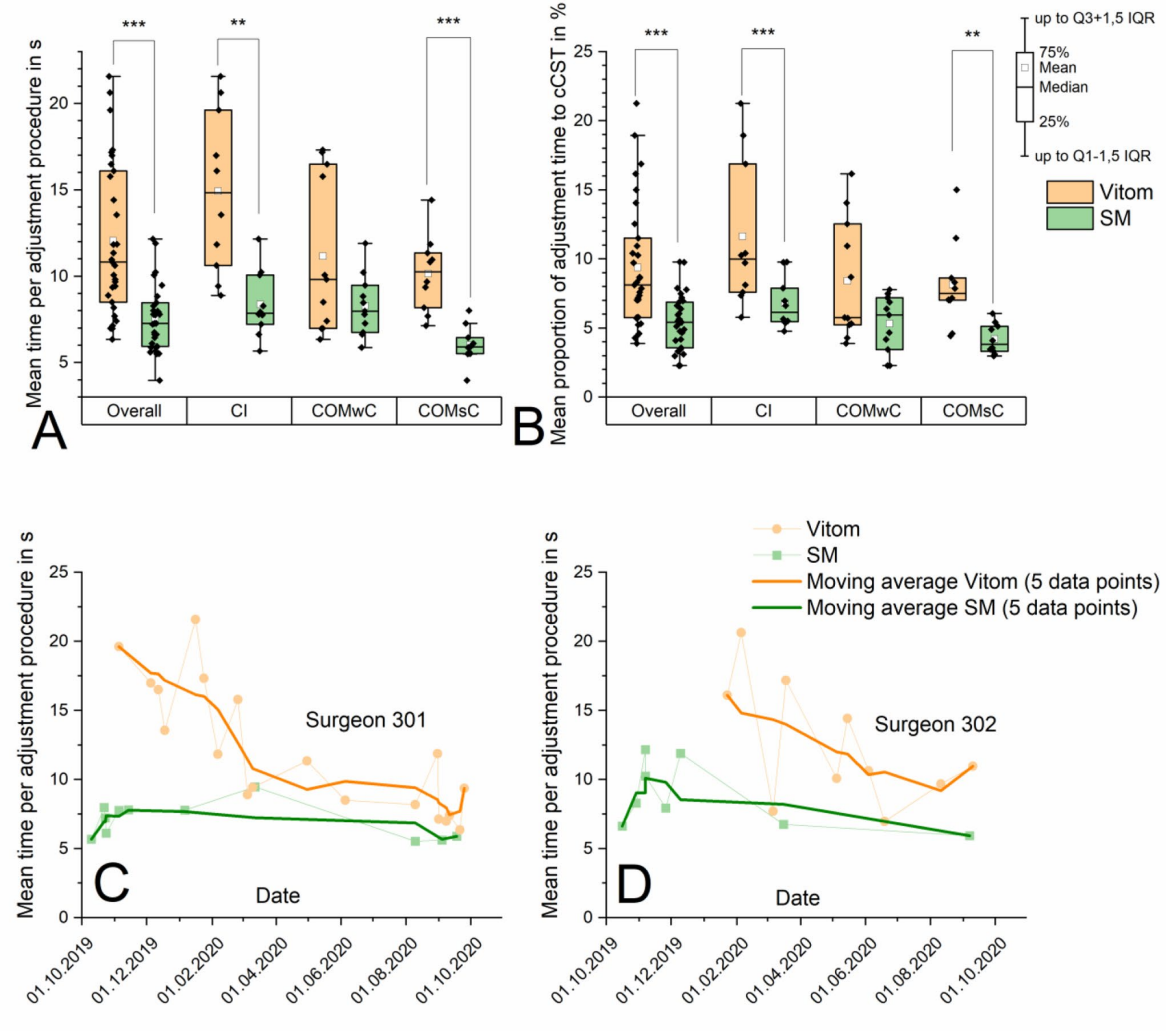
**A/B** Mean time per adjustment procedure **A** and proportion of adjustment time to cCST **B** significant prolongation with the Vitom in the pooled data set (‘overall’) and in the subgroups ‘CI’ and ‘COMsC’. **C/D** Learning curves of surgeons 301 **C** and 302 **D** for all procedures with the Vitom and the SM based on the parameter ‘cCST’ compared to the use of the SM, the moving average indicates a learning progress with the Vitom with decreasing times over the course of the study. In contrast, graphs of the SM group do not indicate a clear learning curve. * *p* < 0.05, ** *p* < 0.01, *** *p* < 0.001

**Fig. 5:**
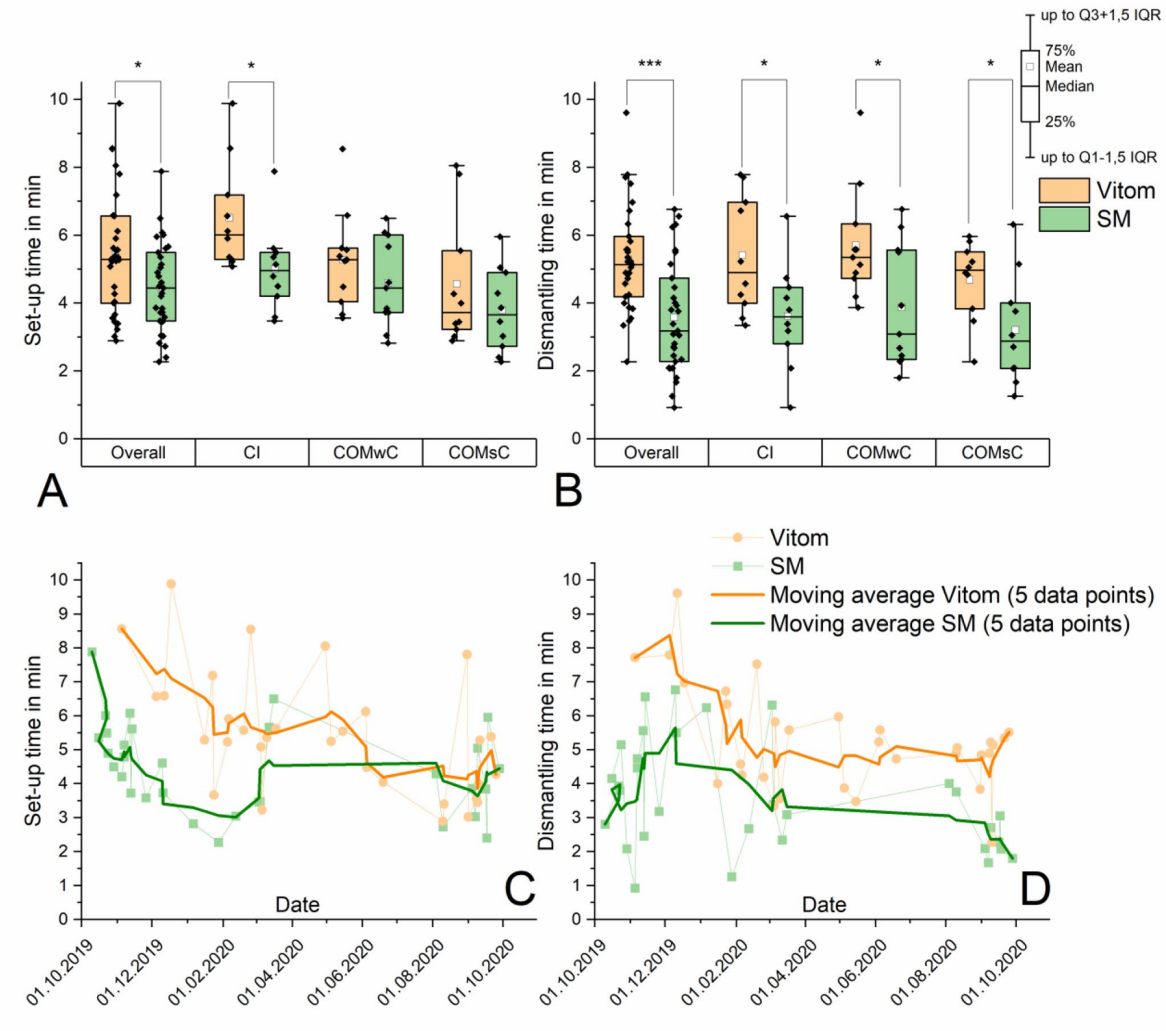
**A** Set-up time: significant prolongation with the VITOM in the pooled data set (‘overall’) as well as in the subgroup ‘CI’. **B** Dismantling time: significant prolongation with the VITOM in all groups including the subgroups. **C/D** Learning curve for set-up and dismantling of the VITOM and the SM across all procedures. Moving average indicates a learning progress with the VITOM with decreasing times over the course of the study. In contrast, graph of the SM procedures do not indicate a typical shape of a learning curve

The learning effect during the course of the study was assessed using the parameters ‘cCST’ (surgeon 301, only CI operations, Fig. 3D), ‘mean time per adjustment procedure’ (surgeons 301 and 302, data pool of all procedures, Fig. 4C/D), ‘set-up time’ and ‘dismantling time’ (data pool of all procedures, Fig. 5C/D) for both visualization systems. To determine the learning curve, the moving average across 5 corresponding single data points was calculated.

### Statistical analysis

Statistical analyses were conducted using IBM SPSS^®^ Statistics 27 (IBM, Armonk, USA) and Origin (OriginLab, Northampton, U.S.). For all analyses, a significance level of 5.0% was chosen, ensuring that results were marked as significant with *p* ≤ 0.05 in the hypothesis testing. The p values in the graphs are indicated as follows: * *p* < 0.05 (significant), ** *p* < 0.01 (highly significant), and *** *p* < 0.001 (highly significant). Values are presented as mean ± standard deviation. If the running text refers to a tendency, the data merely show a statistically non-significant trend. When the terms ‘overall’ or ‘total’ are used in the text, they refer to the general comparison of the two study arms ‘VITOM’ vs. ‘surgical microscope’, without analyses in the subgroups ‘CI’, ‘COMwC’ or ‘COMsC’.

All data of the different subgroups included in the SM and the exoscope study arm were tested for normal distribution by means of the Kolmogorov-Smirnov and Shapiro-Wilk test. Normal distribution could not be confirmed in several cases, Therefore the nonparametric Mann-Whitney-U-test was applied for further comparative statistical analyses of the data between the study arms.

## Results

Please refer to Annex 2 to receive a tabular analysis regarding the measures of the above-mentioned objective outcome parameters.

### Corrected cut suture time (please refer to Fig. 3A)

The application of the VITOM resulted in a significant prolongation of the overall average **cCST** by 25.7% compared to the SM (106.9 ± 45.8 min _E+_ vs. 85.2 ± 34.0 min _E−_; *p* < 0.05).

Subgroup analyses showed heterogeneous results regarding the extent of prolongation. In the COMsC subgroup, the application of the VITOM resulted in a tendential mean prolongation by 3.3% (104.1 ± 53.3 min _E+_ vs. 100.8 ± 45.7 min _E−_; *p* > 0.05). In the CI subgroup, the tendential mean prolongation was approximately 15.7% (84.6 ± 20.8 min _E+_ vs. 73.1 ± 19.9 min _E−_; *p* > 0.05) whereas the COMwC subgroup displayed a significant mean prolongation of 58.0% (129.6 ± 47.8 min _E+_ vs. 82.0 ± 28.8 min _E−_; *p* < 0.05).

### ‘Pure’ operating time (please refer to Fig. 3B)

The results regarding the overall average **POT** tended to be comparable to the results of the corrected CST with a tendential prolongation of 21.1% in the VITOM study arm (97.6 ± 44.5 min _E+_ vs. 80.6 ± 33.6 min _E−_; *p* > 0.05). However, the differences did not meet statistical significance.

Subgroup analyses displayed heterogeneous results again regarding the extent of prolongation. In the COMsC subgroup the application of the VITOM did not prolong operating time significantly (96.1 ± 49.9 min _E+_ vs. 96.7 ± 44.1 min _E−_; *p* > 0.05). In the CI subgroup, the tendential mean prolongation was 9.1% (74.5 ± 19.1 min _E+_ vs. 68.3 ± 19.5 min _E−_; *p* > 0.05), whereas the COMwC subgroup displayed a significant mean prolongation of 55.7% (119.9 ± 47.8 min _E+_ vs. 77.0 ± 29.3 min _E−_; *p* < 0.05).

### Adjustment time per surgery, mean time per adjustment procedure and proportion of the adjustment time to the corrected cut suture time (please refer to Figs. 3C and 4A and B)

Comparing both study arms, the application of the VITOM resulted in a significantly increased **mean total adjustment time per surgery** (9.2 ± 4.1 min _E+_ vs. 4.3 ± 1.6 min _E−_; *p* < 0.001) as well as in their subgroups compared to the application of the SM (CI: 9.7 ± 4.8 min _E+_ vs. 4.8 ± 1.0 min _E−_; *p* < 0.001; COMwC: 9.8 ± 3.6 min _E+_ vs. 4.1 ± 1.9 min _E−_; *p* < 0.01; COMsC: 8.0 ± 4.0 min _E+_ vs. 4.1 ± 1.9 min _E−_; *p* < 0.001). The same applies to the **mean time per adjustment procedure** (12.0 ± 4.4 s _E+_ vs. 7.6 ± 1.9 s _E−_ vs.; *p* < 0.001). Subgroup analyses here could confirm for both CI and COMsC subgroups significant differences (CI: 14.9 ± 4.7 s _E+_ vs. 8.4 ± 1.9 s _E−_; *p* < 0.01; COMsC: 10.1 ± 2.2 s _E+_ vs. 6.0 ± 1.1 s _E−_; *p* < 0.01). In the COMwC subgroup only a tendency without significance could be observed (11.2 ± 4.5 s _E+_ vs. 8.3 ± 1.8 s _E−_; *p* > 0.05). The VITOM led to a significant overall increase of 74.1% compared to the SM resulted regarding the **proportion of adjustment time to cCST** (9.4 ± 4.5% _E+_ vs. 5.4 ± 2.0% _E−_; *p* < 0.001). Subgroup analyses identified the largest differences in the COMsC subgroup, where the proportion was almost twice as large (+ 95.2%) for the exoscope as for the SM (8.2 ± 3.1% _E+_ vs. 4.2 ± 1.1% _E−;_*p* < 0.01). There was also a significant difference (+ 70.6%) in the CI group (11.6 ± 5.4% _E+_ vs. 6.8 ± 1.8% _E−_; *p* < 0.001). A trend was also confirmed in the COMwC subgroup (+ 58.5%), however, the differences failed to reach significance (8.4 ± 4.3% _E+_ vs. 5.3 ± 2.0% _E−_; *p* > 0.05).

### Set-up and dismantling time (please refer to Fig. 5A and B

As all surgical procedures with the VITOM and the SM, respectively, required the same surgical set-up. Both set-up time (5.4 ± 1.8 min _E+_ vs. 4.5 ± 1.3 min _E−_; *p* < 0.05) and dismantling time (5.3 ± 1.5 min _E+_ vs. 3.6 ± 1.6 min _E−_; *p* < 0.001) lasted significantly longer with the VITOM than with the SM overall and in subgroups.

### Learning curves

Two surgeons (surgeon ID “301” and “302”) who are both experts in their field with over 20 years of surgical experience, performed 77.4% of all surgical procedures. They also conducted 29 of 31 procedures with the exoscope. Therefore, looking at their learning curves may be beneficial to gain valuable findings. Surgeon “301” performed a total of 30 surgeries, still 12 more than surgeon “302” (*n* = 18).

### Corrected cut suture time (please refer to Fig. 3D)

Regarding the **cCST** for the subgroup CI procedures with the VITOM, a learning curve could only be illustrated for surgeon ‘301’ (*n* = 7), as the number of cases for surgeon ‘302’ was too low. Furthermore, we only present the results of CI surgery, as this procedure involves a more standardized surgical technique than COMsC and COMsC procedures.

The time averaged across the first 3 procedures was 80.0 min, and 63.1 min across the last 3 procedures. This corresponded to a reduction of 21.1%. Although there were only four measured values for the SM-group, the mean cCST was significantly lower with the SM than with the exoscope (75.8 ± 16.9 min _E+_ vs. 56.7 ± 6.1 min _E−_; *p* < 0.05) despite the learning curve.

### Adjustment times per adjustment procedure (please refer to Fig. 4C/D)

For both surgeons (surgeon ‘301’ and ‘302’), a learning curve could be shown regarding the adjustment time when using the exoscope with a trend of decreasing average time per adjustment. Comparing the first 3 procedures (surgeon 301: 16.5 s to19.6 s; surgeon 302: 7.7 s to 16.1 s) to the last 3 procedures (surgeon 301: 7.4 s to 9.4 s; surgeon 302: 7.0 s to 11.0 s), the average adjustment time decreased by approximately 47.2% for both surgeons. The trend in the SM group showed a flatter course and a smaller spread of the measurements than in the exoscope group for both surgeons. The learning curve of the exoscope group tends to approach that of the SM group over the course of the study and, in the case of the surgeon 301, nearly overlaps it by the end of the study.

### Set-up and dismantling time (please refer to Fig. 5C/D)

The pooled data across all procedures of all surgeons indicated a learning curve for the exoscope. A reduction in times could also be displayed for the SM groups, however the graph did not show the typical shape of a learning curve. For the first five procedures, preparation time and dismantling time averaged 7.4 min and 7.2 min, respectively, in the exoscope study arm as well as 5.9 min and 4.0 min, respectively, in the SM study arm. For the last five procedures, preparation time and dismantling time averaged 4.5 min and 4.7 min, respectively, in the exoscope study arm as well as 4.3 min and 2.4 min, respectively, in the SM study arm. This corresponded to a reduction regarding the preparation time of 39.2% (VITOM group) and 27.1% (SM group) as well as of 34.7% (VITOM group) and 40.0% (SM group) regarding the dismantling time. The graph of the VITOM group approaches the curve of the SM group in terms of set-up time, but not in terms of dismantling time.

## Discussion

### Is the trend towards a prolongation of the overall mean cCST and mean POT when performing surgery with the VITOM compared to the SM just a matter of the presence of a learning curve?

There are various explanations for the prolongation of the overall average cCST and the increase of the mean POT for all subgroups when using the VITOM. A key aspect associated with any new system that is applied for the first time is the existence of a **learning curve**. Especially for the CI surgeries, where surgeon 301 performed most of the operations, our data indicated the presence of such a learning curve. The tendency of a reduction of the cCST and thus the surgical time over the period of the study could be observed (Fig. 3D). In the learning curve, a comparable cCST of the VITOM to the SM was seen in the fifth operation of surgeon 301, so the differences in CST should be less significant with longer observation periods. Further studies with a longer observation period would be needed to determine whether cCST and POT differ significantly from SM when using the VITOM after reaching the steady state of the learning curve.

When differentiating between the subgroups, the VITOM surgeries took longer (CI: +11.5 min; COMsC: +3.3 min) on average than with the SM. A significant difference with a mean prolongation of the cCST of + 47.6 min when using the VITOM was only found for COMwC. A possible explanation could be the sometimes **higher complexity of the procedures**, as the expansion of a cholesteatoma can vary. Differences in the handling or visualization of the two systems - both subjective parameters to be presented in a future paper - can contribute significantly to the reduction or extension of operating times as the level of difficulty of the operation increases. In contrast, the surgical procedures for COMsC and especially for CI operations are **standardized** [17]. In these cases, the influence of the VITOM on the cCST does not seem to have been strong enough to result in a significant difference.

Regarding the POT, there was again a significant increase of 42.9 min with the VITOM only for COMwC. The results tend to be comparable to those of Colombo et al. and Rubini et al. as there were no significant differences in the operating time between the two visualization systems [7, 12]. However, Rubini et al. found a shorter average surgical time, which was only the case for COMsC (96.1 ± 49.9 min _E+_ vs. 96.7 ± 44.1 min _E−_) in this study. One possible reason is the smaller sample size in Rubini et al. (*n*_Rubini_ = 24 vs. *n* = 62) and the differences in the type of procedures, mainly lateral skull base procedures.

### Repeated use of the VITOM led to a learning curve affecting the adjustment time and the proportion of the adjustment time to cCST

The adjustment times, as well as the adjustment times per adjustment process and the proportion of the adjustment time to the corrected CST, were significantly increased with the VITOM compared to the SM. However, **learning effects** tended to reduce these times during the study period. At the beginning of the study, the average adjustment time per adjustment process of the first 3 surgeries amounted to a maximum of 17.7 and 14.8 s for operators 301 and 302 with the VITOM and decreased towards the end (last 3 surgeries) to a maximum of 7.7 and 9.2 s which is a reduction of 56.5% and 37.8%. Unlike the SM, the VITOM is a relatively new 3D visualization system. Both surgeons who performed most of the procedures had been experienced with the SM for more than 20 years and the adjustment processes had already reached a stable state. This was not the case with the VITOM.

Another reason may be **the separation of the exoscope set-up** into a camera unit above the surgical field and a control unit, requiring the adjustment of two physically separate components. In contrast, the SM integrates these components into a single structural unit, wherein adjustment functions are accessible via both handles. Frequently, multiple adjustments were required for the VITOM to achieve the intended visual perspective, as the adjustment procedures were not as ingrained as those for traditional SMs. Ally et al. similarly noted challenges in operating the main control button, which could lead to difficulties in achieving the desired image settings [11]. The corresponding frequent readjustment can thus disrupt the operation flow [11, 18]. Furthermore, Minoda and Miwa described the repeated refocusing with a separate controller as uncomfortable [9].

### Influence of subgroup size and learning curves on the interpretation of results

The interpretation of the own data, especially in the subgroups, is limited by two further confounding factors: sample size and learning curve. Based on the initial sample size calculation (*n* = 27 per study arm), the study is underpowered for the final assessment of the subgroup results (CI, COMsC and COMwC). The design used to assess subgroup results therefore increases the likelihood of false negatives. Therefore, a final judgment on whether VITOM 3D is actually inferior to SM for each of the above types of intervention can only be made by further studies with an increased subgroup size.

The learning curves (Figs. 3D, 4C/D and 5 C/D) show a promising high level of training effect when using the VITOM. In all evaluated learning curves (corrected cut suture time, CI surgery Fig. 3D; adjustment time per adjustment procedure, all procedures Fig. 4C/D; set-up and dismantling time Fig. 5C/D), the results obtained with the VITOM approach those of the SM and even intersect them (corrected cut suture time (surgeon 301), mean time per adjustment procedure (surgeon 301), set-up time). In the long term, it might be likely that the evaluated objective parameters of both systems (VITOM and SM) converge. However, this assumption remains speculative on the basis of the current investigations. Unfortunately, the learning effect also appears to be confounder in our own data. Due to the lack of possibility for exclusion, it influences and increases the spread of the data between the VITOM and SM groups. This also implies the necessity of follow-up studies with a higher number of patients per surgeon in order to exclude learning effects with statistical methods and to be able to make a final statement about the superiority or inferiority of VITOM compared to SM.

### Limitations of the VITOM 3D for routine application in ear surgery - own results compared to current literature

Please refer to Table 1 to receive an overview of the current literature data in comparison to our own results. Although the discussion of subjective parameters will take place in the second part of the paper, they cannot be entirely omitted here as they impact objective parameters, such as extended operating times with the VITOM. Challenges include inadequate brightness control, limited depth perception, poor optical representation, and issues with contrast and resolution. Handling challenges, such as manual repositioning and lack of continuous zoom, were also noted. These findings align with those reported in other studies, including Wierzbicka et al. (2021), Ally et al. (2021), Colombo et al. (2021), and others, who identified similar issues with brightness regulation, image quality, and handling difficulties. Extended operating times were also a common observation. These findings will be discussed in detail in the second part of the paper.

**Table 1:** Limitations of exoscopes at a glance stratified by authors; white background: VITOM, gray background: Mode V™

	Our data, 2024	Wierzbicka et al., 2021	Ally et al., 2021	Colombo et al., 2021	Rubini et al., 2020	Smith et al., 2019	Minoda & Miwa, 2019	Garneau et al., 2019
**Deficiencies regarding the visualization of the field of view**								
Regulation of brightness	x		x	x	x			
Insufficient contrast and resolution	x	x		x	x			
Insufficient illumination of surgical sites entered via narrow access routes	x	x		x	x	x		
Insufficient image quality at magnification	x	x		x		x	x	
Insufficiencies in the natural color display	x			x				
Insufficient depth impression	x	x						x
Insufficient depth of field / size of field of view	x		x				x	
Image transmission latency	x							
**Insufficiencies in the handling of the system**								
Holder/optical unit: challenges in repositioning	x		x					
Controller (IMAGE1 PILOT): exaggerated sensitivity of the main control button			x					
Lack of a continuous zoom mode	x							
**Changes of the workplace atmosphere during the surgery**								
Potential risk of distraction	x			x				
Extended operating time/ time delay	x			x				x

## Summary

This study provides a prospective and systematic comparison between the application of an exoscope (VITOM 3D) and SM in reconstructive middle ear surgery (COMsC and COMwC) and CI surgery. Both visualization systems have been compared regarding intraoperative objective measurement parameters (e.g. cut suture time, corrected cut suture time, pure operating time, adjustment time, set up and dismantling time) and subjective ratings of the visual and handling quality (data part of a future paper). In summary, the VITOM 3D was inferior to the SM in all subgroups regarding the intraoperative objective measurements and the subjective ratings of the visual and handling quality. Mean cCST as well as set-up times were prolonged by more than 20.0% with the VITOM. Dismantling times increased by as much as 47.2%, while adjustment times per adjustment procedure increased by 57.9% and the ratio of adjustment time to cCST increased by 74.1%. However, the results showed the presence of a learning curve. At the end of the study, the objective measurements of the VITOM approached those of the SM in some categories. Further studies with a longer observation period would be needed to determine whether cCST and POT differ significantly from SM when using the VITOM after reaching the steady state of the learning curve. A future paper will present the subjective parameters. By summarizing the objective parameters presented here and the subjective parameters presented later, it will be possible to make suggestions for the further development of the VITOM, the effectiveness of which will need to be re-evaluated in future studies in order to reduce the current technical gap between current exosocopes and SM.

## Electronic supplementary material

Below is the link to the electronic supplementary material.

Supplementary Material 1

## Data Availability

In this study, all relevant processed data are within the paper and its supporting figures, tables and annexes. Raw data is available on request.

## Declarations

### Permission to reproduce material from other sources

All materials and content reproduced from other sources have been appropriately credited. Permissions have been obtained for all copyrighted materials used in this manuscript and are documented in the supplementary materials.
