# Supplementary material for: Prospective study of the application of a 3D exoscope system (VITOM 3D) in ear surgery compared to conventional surgical microscopes: part I - analysis of objective parameters
Source: Eur Arch Otorhinolaryngol. 2024 Nov 22;282(5):2263–74. doi: 10.1007/s00405-024-09096-9 (PMC12055886; doi:10.1007/s00405-024-09096-9)
Supplement: Supplementary file 1 — Supplementary Material 1 [file 405_2024_9096_MOESM1_ESM.docx]

**Annex 1** Demographics of the patients included in the study


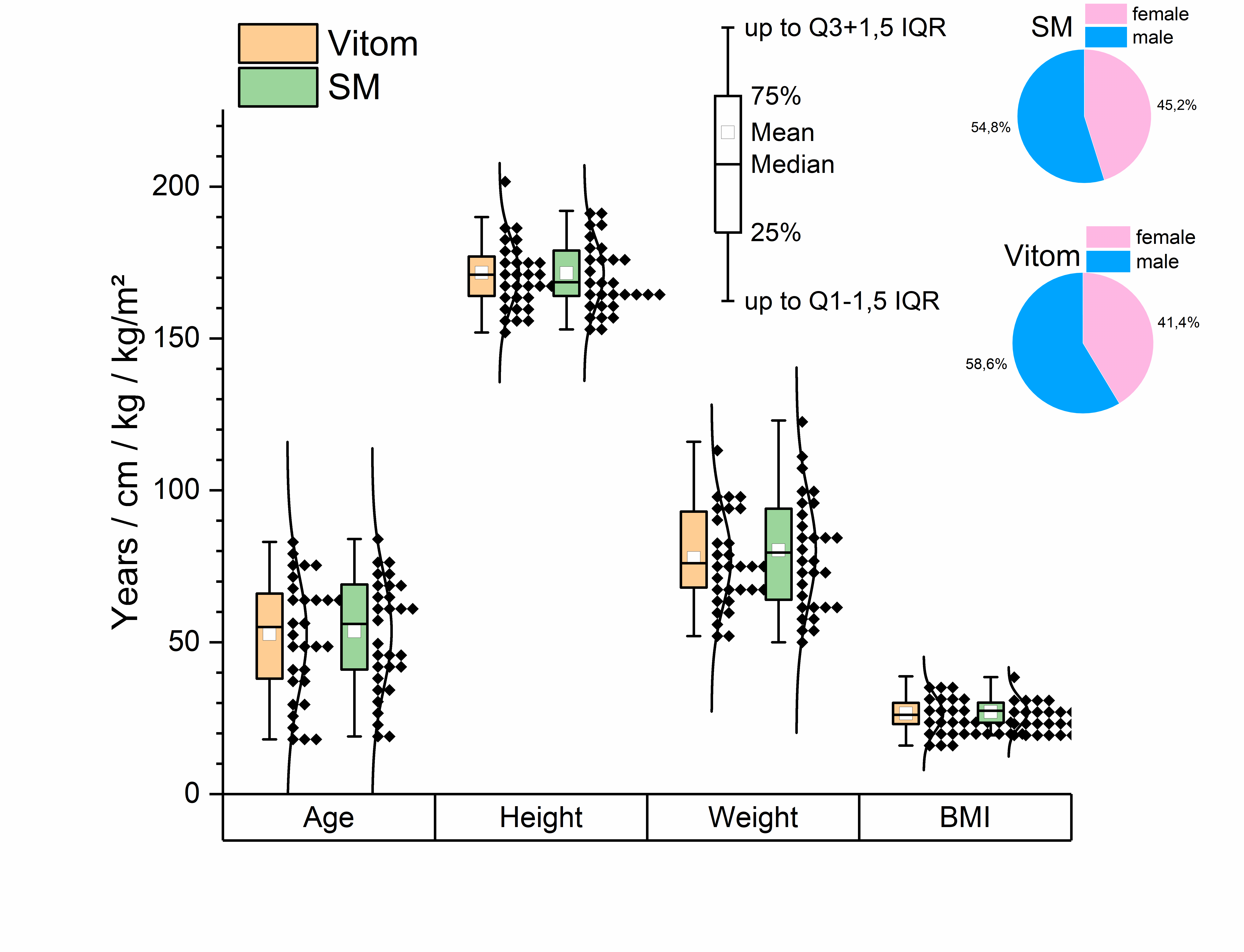


**Annex 2** Tabular analysis regarding the objective outcome parameters

|  |  | **Overall** | **CI** | **COMwC** | **COMsC** |
| --- | --- | --- | --- | --- | --- |
| **Corrected CST [min]** | Overall | 96.0 ± 41.5 (*n*= 62) [85.5; 106.6] | 78.8 ± 20.7 (*n*= 20) [69.2; 88.5] | 105.8 ± 45.6 (*n*= 22) [85.6; 126.0] | 102.5 ± 48.3 (*n*= 20) [79.8; 125.1] |
|  | SM | 85.2 ± 34.0* (*n*= 31) [72.7; 97.7] | 73.1 ± 19.9 (*n*= 10) [58.8; 87.4] | 82.0 ± 28.8* (*n*= 11) [62.6; 101.4] | 100.8 ± 45.7 (*n*= 10) [68.1; 133.5] |
|  | VITOM | 106.9 ± 45.8* (*n*= 31) [90.1; 123.7] | 84.6 ± 20.8 (*n*= 10) [69.7; 99.5] | 129.6 ± 47.8* (*n*= 11) [97.6; 161.7] | 104.1 ± 53.3 (*n*= 10) [66.0; 142.2] |
| **Pure operating time [min]** | Overall | 89.1 ± 40.0 (*n*= 62) [78.9; 99.2] | 71.4 ± 19.1 (*n*= 20) [62.5; 80.3] | 98.4 ± 44.5 (*n*= 22) [78.7; 118.2] | 96.4 ± 45.9 (*n*= 20) [74.9; 117.9] |
|  | SM | 80.6 ± 33.6 (*n*= 31) [68.3; 92.9] | 68.3 ± 19.5 (*n*= 10) [54.3; 82.2] | 77.0 ± 29.3* (*n*= 11) [57.3; 96.7] | 96.7 ± 44.1 (*n*= 10) [65.2; 128.3] |
|  | VITOM | 97.6 ± 44.5 (*n*= 31) [81.2; 113.9] | 74.5 ± 19.1 (*n*= 10) [60.8; 88.1] | 119.9 ± 47.8* (*n*= 11) [87.8; 152.0] | 96.1 ± 49.9 (*n*= 10) [60.3; 131.8] |
| **Adjustment time [min**] | Overall | 6.8 ± 3.9 (*n*= 62) [5.8; 7.7] | 7.2 ± 4.2 (*n*= 20) [5.2; 9.2] | 7.0 ± 4.0 (*n*= 22) [5.2; 8.7] | 6.1 ± 3.6 (*n*= 20) [4.4; 7.8] |
|  | SM | 4.3 ± 1.6*** (*n*= 31) [3.7; 5.0] | 4.8 ± 1.0** (*n*= 10) [4.1; 5.4] | 4.1 ± 1.9*** (*n*= 11) [2.8; 5.4] | 4.1 ± 1.9* (*n*= 10) [2.8, 5.4] |
|  | VITOM | 9.2 ± 4.1*** (*n*= 31) [7.7; 10.7] | 9.7 ± 48** (*n*= 10) [6.2; 13.1] | 9.8 ± 3.6*** (*n*= 11) [7.4; 12.2] | 8.0 ± 4.0* (*n*= 10) [5.2; 10.9] |
| **Adjustment times per adjustment procedure [s]** | Overall | 9.8 ± 4.0 (*n*= 62) [8.8; 10.8] | 11.6 ± 4.9 (*n*= 20) [9.4; 14.0] | 9.7 ± 3.7 (*n*= 22) [8.1; 11.3] | 8.1 ± 2.7 (*n*= 20) [6.8; 9.3] |
|  | SM | 7.6 ± 1.9*** (*n*= 31) [6.9; 8.3] | 8.4 ± 1.9** (*n*= 10) [7.0; 9.7] | 8.3 ± 1.8 (*n*= 11) [7.1; 9.5] | 6.0 ± 1.1*** (*n*= 10) [5.2; 6.8] |
|  | VITOM | 12.0 ± 4.4*** (*n*= 31) [10.4; 13.7] | 14.9 ± 4.7** (*n*= 10) [11.5; 18.3] | 11.2 ± 4.5 (*n*= 11) [8.1; 14.2] | 10.1 ± 2.2*** (*n*= 10) [8.6; 11.7] |
| **Proportion of adjustment time to corrected CST [%]** | Overall | 7.4 ± 4.0 (*n*= 62) [6.4; 8.4] | 9.2 ± 4.6 (*n*= 20) [7.0; 11.4] | 6.9 ± 3.7 (*n*= 22) [5.2; 8.5] | 6.2 ± 3.1 (*n*= 20) [4.7; 7.6] |
|  | SM | 5.4 ± 2.0*** (*n*= 31) [4.7; 6.1] | 6.8 ± 1.8*** (*n*= 10) [5.5; 8.1] | 5.3 ± 2.0 (*n*= 11) [3.9; 6.7] | 4.2 ± 1.1** (*n*= 10) [3.4; 5.0] |
|  | VITOM | 9.4 ± 4.5*** (*n*= 31) [7.7; 11.0] | 11.6 ± 5.4*** (*n*= 10) [7.8; 15.5] | 8.4 ± 4.3 (*n*= 11) [5.5; 11.3] | 8.2 ± 3.1** (*n*= 10) [6.0; 10.4] |
| **Set-up time [min]** | Overall | 5.0 ± 1.6 (*n*= 62) [4.5; 5.4] | 5.8 ± 1.6 (*n*= 20) [5.0; 6.5] | 4.9 ± 1.4 (*n*= 22) [4.3; 5.5] | 4.2 ± 1.6 (*n*= 20) [3.4; 4.9] |
|  | SM | 4.5 ± 1.3* (*n*= 31) [4.0; 5.0] | 5.0 ± 1.3* (*n*= 10) [4.1; 6.0] | 4.6 ± 1.3 (*n*= 11) [3.7; 5.5] | 3.8 ± 1.2 (*n*= 10) [3.0; 4.7] |
|  | VITOM | 5.4 ± 1.8* (*n*= 31) [4.8; 6.1) | 6.5 ± 1.6* (*n*= 10) [5.4; 7.7] | 5.3 ± 1.4 (*n*= 11) [4.3; 6.2] | 4.6 ± 1.9 (*n*= 10) [3.2; 6.0] |
| **Dismantling time [min]** | Overall | 4.4 ±1.8 (*n*= 62) [4.0; 4.9] | 4.5 ± 1.8 (*n*= 20) [3.6; 5.4] | 4.8 ± 1.9 (*n*= 22) [3.9; 5.6] | 3.9 ± 1.6 (*n*= 20) [3.2; 4.7] |
|  | SM | 3.6 ± 1.6*** (*n*= 31) [3.0; 4.2] | 3.6 ± 1.5* (*n*= 10) [2.5; 4.7] | 3.9 ± 1.8* (*n*= 11) [2.7; 5.1] | 3.2 ± 1.6* (*n*= 10) [2.1; 4.4] |
|  | VITOM | 5.3 ± 1.5*** (*n*= 31) [4.7; 5.8] | 5.4 ± 1.7* (*n*= 10) [4.2; 6.6] | 5.7 ± 1.6* (*n*= 11) [4.6; 6.8] | 4.7 ± 1.2* (*n*= 10) [3.9; 5.5] |

**Annex 2** Means, standard deviations and 95% confidence interval (angular brackets) of corrected cut suture time (CST), pure operating time, adjustment time, adjustment times per adjustment procedure, percentage of adjustment time to corrected CST, set-up time and dismantling time stratified by visualization system (VITOM and surgical microscope (SM)) and type of procedure: CI (cochlear implant), COMwC (chronic otitis media with cholesteatoma) and COMsC (chronic otitis media without cholesteatoma), n stands for the number; significant difference between methods (* *p* < 0.05, ** *p* < 0.01, *** *p* < 0.001)
